# D5 digital circular workflow: five digital steps towards matchmaking for material reuse in construction

**DOI:** 10.1038/s44296-024-00034-8

**Published:** 2024-11-01

**Authors:** Catherine De Wolf, Brandon S. Byers, Deepika Raghu, Matthew Gordon, Vanessa Schwarzkopf, Eleftherios Triantafyllidis

**Affiliations:** Circular Engineering for Architecture, Department of Civil, Environmental and Geomatic Engineering, ETH Zurich, Zurich, Switzerland

**Keywords:** Civil engineering, Carbon and energy

## Abstract

The intersection of digital transformation and circular construction practices presents significant potential to mitigate environmental impacts through optimised material reuse. We propose a five-step (D5) digital circular workflow that integrates these digital innovations towards reuse, validated through real-world case studies. We assessed a variety of digital tools for enhancing the reuse of construction materials, including digital product passports, material classification assisted by artificial intelligence (AI), reality capture, computational design, design inspired by generative AI, digital fabrication techniques, extended reality, and blockchain technology. Using action research through a multiple case study approach, we disassembled several buildings that were set for demolition and subsequently designed and executed construction projects using the salvaged materials. Our findings indicate that digital transformation for detection, disassembly, distribution, design, and finally deployment significantly support the application of circular economy principles. We demonstrate the potential of the proposed workflow for industry implementation and scalability.

## Introduction

New digital methods have the potential to rapidly enhance the circularity of the entire architecture, engineering, and construction (AEC) sector—a sector that faces the significant challenge of meeting global housing needs while reducing its 37% contribution to greenhouse gas emissions^1^. Construction alone is responsible for 50% of all material consumption and more than a third of all solid waste^2^.

One of the main reasons for the sector’s high amounts of waste is that we construct buildings following a linear model: extracting, producing, using, and then disposing of materials and resources. A circular model is needed to derive maximum value from building materials by reusing them at the end of their service life as new resources. A circular economy^3–6^ promotes a regenerative system where repair, reuse, recycling, and energy recovery minimise resource use and emissions^7^. More specifically, the reuse of materials in the built environment is essential to tackle challenges such as the scarcity of resources, waste treatment, and the climate crisis. Indeed, the Intergovernmental Panel on Climate Change (IPCC) encourages material reuse as a near-term response to climate change^8^, while the United Nations Environment Programme (UNEP) aims for near-zero emissions in the building sector by 2030^9^. Implementing circular principles benefits the environment, economy, and society^10–18^.

While pioneering circular building projects^19–22^ and innovative reuse platforms and marketplaces for construction^23–29^ have shown promise in localised settings, global implementation on a large scale has not yet been successful. Material recovery remains labour-intensive, with experts manually measuring and cataloguing data for material reuse. Building projects typically involve complex, multifaceted challenges that are tackled by numerous stakeholders operating in isolated silos. As a result, circular strategies are not yet broadly applied in construction practice. Digital transformation can fill this gap by streamlining these processes and enhancing efficiency.

However, the construction industry remains the least digitised sector due to its fragmentation and risk aversion, thus struggling to attract digital talent and impeding its adoption of digital technologies^30,31^. Research in circular building practices^32^ highlights challenges that digital technologies can help overcome. Frameworks for digital technologies towards circular construction have been developed for sustainable construction using specific materials, such as upcycled wood^33^, or through literature reviews^32,34^. Yet, entire workflows of digital innovations for material reuse have rarely been integrated and validated on a full building scale. Therefore, a viable digital circular construction management system for building material reuse needs to be developed. In the context of a Circular Digital Built environment framework (CDB)^32^ and a review of existing digital technologies for a circular built environment^34^, we assessed the effectiveness of the proposed digital technologies in facilitating material reuse in actual building projects.

In this paper, we propose a five-step workflow for digital transformation towards materials’ reuse, which we refer to as a “D5 Digital Circular Workflow” (D5 Workflow) for integrating digital tools in assessing existing buildings, in their disassembly of reusable materials for distribution, and in their design and construction with the salvaged materials. Employing a multi-case study action research approach, we evaluate the impact and effectiveness of integrating digital tools into circular construction. Digital technologies are applied to different test cases in each of the five steps to validate the workflow. Our research focuses on case studies that helped us analyse how digital technologies can facilitate deconstruction to reuse materials in construction. Aligning the logistics of disassembling buildings with constructing new ones is challenging. To make material reuse more effective, the end-of-life of buildings set for demolition should be connected to the start-of-life of new buildings, minimising storage times. Although platforms exist for listing reused materials, finding the right match remains a challenge, connecting architects, contractors, facility managers, and certifiers skilled in working with reused materials^35^.

Our workflow introduces a comprehensive digital integration to enhance this “matchmaking” process, which systematically pairs reclaimed materials (supply) with appropriate construction projects (demand) by connecting AEC stakeholders (matching). To create effective matches, construction stakeholders need to know who has waste materials to offer, where they can store them, and who wants to turn them into resources. This involves ensuring that materials salvaged from demolition sites are effectively identified, catalogued, and aligned with the specific needs and requirements of building projects. This process is structured into a five-step approach, each beginning with the letter ‘D’—hence the term “D5 workflow.” This workflow incorporates advanced digital tools across these five critical stages:

1. **Detection**: Use urban data combined with machine learning (ML) and computer-vision (CV) algorithms to identify sites suitable for material reuse and incorporate these materials into building information modelling (BIM) systems.
2. **Disassembly**: Catalogue materials extensively, employing reality capture, scan-to-BIM and CV to enable robotics and extended reality (XR) for disassembly.
3. **Distribution**: Create digital product passports (DPPs) to efficiently track, trace, and trade materials from demolition to new construction sites.
4. **Design**: Apply generative Artificial Intelligence (AI) and computational design algorithms to create and match designs with reclaimed materials.
5. **Deployment**: Use subtractive and additive manufacturing to integrate bespoke reclaimed elements, assembling them in new constructions with XR techniques.

**Detection** of suitable materials for reuse can be enabled by using ML and CV algorithms. For the effective mapping of the environmental impacts of building materials, various systems, such as Geographical Information Systems (GIS), BIM, and construction management software must exchange and use information^36^. In the context of identifying reuse patterns, advancements in ML and CV have significantly improved the interoperability of data representations. This enhancement supports the spatial and temporal mapping of existing public housing stocks on an urban scale, thereby enabling the scaling of applications to entire cities^37,38^. In addition, these technologies facilitate the efficient matching of available material resources (supply) with the material needs for new construction that incorporate reused materials (demand). ML, particularly through deep learning^36–38^, has been used extensively in other sectors to process data and recognise patterns. Recently, this technology has been applied in the construction industry to process large, unstructured datasets such as building demolition records for forecasting the amount of salvage and waste materials obtainable at the end-of-life of buildings^39^. CV is also increasingly being applied to construction sites to enhance operations, such as building inspections. For example, research and industry projects use imagery from various sources such as unmanned aerial vehicles (UAVs), satellite imagery, social media, public webcams, and capturing cars (e.g. Google Street View)^40–42^. To fully leverage this technology for circular construction, global datasets such as cadastral information^43^ and imagery data must be integrated to forecast reusable building materials.

During **disassembly**, scan-to-BIM has enabled the cataloguing of materials for reuse. AEC firms use BIM to store information in three-dimensional (3D) models of their building projects, e.g., the material types, schedule, and cost^30,44^. BIM is also used to create a repository of materials for the building project, i.e., material passports^45^, capturing information about the type, configuration, volume, and location of materials (similar to DPPs at the product level). However, most existing buildings that will be demolished in the next few decades do not have a BIM model available. Using reality capture techniques, point clouds are often used to create missing BIM models a posteriori. However, processing these point clouds remains difficult due to their unstructured, irregular form^46^. Techniques such as PointNet, adapted from 2D image classification, now facilitate semantic analysis^47–49^, though they often target specific components such as structural steel or scaffolding^50^. CV has also been used on the material scale, for instance, to detect damage such as concrete cracks, steel corrosion, and steel delamination^51^. Moreover, precise data capture techniques have been developed for reusing concrete as dry masonry^52^. Support-vector machine (SVM)-based systems have also helped classify building materials for automated digital reconstruction^53^, but they focus on individual classifications and lack integration with 3D reconstruction. Construction environments present challenges for CV due to their disorderly and cluttered nature. Modern systems often misidentify background details, and active sites contain conditions absent in training sets^54^. Addressing these challenges requires techniques like background randomisation in training sets, selective masking by depth, and analysing common misclassifications to improve accuracy and reliability.

For **distribution**, existing platforms^23^ are designed to capture data about the quantity, quality, location, financial value, and circular utility of materials available for reuse. Increasing efforts from research aim to link material platforms to BIM platforms and integrate product tracking and DPPs into these platforms^32,55^. Blockchain technology is now explored for its potential to enable decentralised data management and enhance transparency and traceability in circular construction^56–59^. Digital platforms commodify buildings as material banks, making building elements tradable and enabling organisations to meet market demands efficiently through economies of scale and scope (e.g., BoKlok^60^, Reflow project^61^). Widely used digital platforms from other sectors (e.g., Amazon, Tinder, AirBnB, Uber) have shown that growth depends on increasing both supply and demand users^30^. Circular platforms should act as catalysts, linking those dealing with building disposal to those starting new constructions. Leveraging digital technology, especially AI algorithms similar to dating app algorithms^62^, could enhance matchmaking in the construction industry. Unlike one-to-one matching systems (e.g., dating apps), a matchmaking service for the reuse of building materials should focus on a many-to-many relationship between reusable building components and potential new constructions, while considering factors like timing, permits, and material characteristics^63^. Tracking and tracing materials in large databases allows for effective matching of available materials for resource allocation.

For the **design**, the potential of unleashing co-creativity between humans and generative AI^64^ is particularly promising^65–67^. Generative AI has the potential to enhance early-phase circular design processes with advanced data management capabilities. A critical component of this shift is handling extensive digital databases that catalogue dismantled building components, allowing architects to effectively match these materials with new design projects. Leveraging AI, specifically through its subset of ML techniques and applying matchmaking algorithms, can further streamline this process and foster an environment where AI augments human creativity in generating innovative design solutions^68^. Generative AI is a subfield of ML and a form of deep learning, which, in addition, uses parts of natural language processing (NLP) for working with natural text in, for instance, Text-to-Image Generators. Next, computational design tools such as parametric design space exploration and rule-based approaches can advance the design process. Optimisation methods, including genetic algorithms, flocking behaviours, or parameter vector manipulation, can be applied within a given design space^69–72^. A bottom-up approach starts with available components and algorithmically assembles them into architectural structures^73–75^. Conversely, a top-down approach commences with a target design and searches the inventory algorithmically^76^. Multi-objective optimisation automates considerations such as feasibility, costs, and impact^77^. Design workflows must be developed to handle diverse material stocks, reduce processing times, and accommodate uniquely shaped reused materials to ensure the best match.

For **deployment**, digital fabrication—a combination of computer-aided design (CAD) data, computer-aided manufacturing (CAM) software, and computer numerical controlled (CNC) hardware for additive and subtractive manufacturing—has been increasingly explored in the AEC industry to produce rapid prototypes, complex elements, and to perform tasks that are repetitive, dangerous, or require precision^78^. In a circular built environment, digital fabrication can be used to design complex connections^79,80^. to make new buildings easier to disassemble^81^. Moreover, XR tools have emerged as an immersive and interactive asset both in the AEC industry and in the broader context of human-centred applications^82–84^. These advancements can benefit the AEC industry and in particular dis- and reassembly processes.

By taking an action research approach, we prototype digital circular techniques on real-world sites. The problem this research aims to address is the need for upscaling sustainable building practices by integrating digital technologies into the circular economy, particularly enhancing material reuse in the construction industry. Moreover, this research aims to strengthen on- and off-site collaboration across the entire value chain towards a circular, low-carbon, zero-waste built environment.

## Results

The action plan employed is to test and improve the technical setup, accuracy, and applicability of our proposed workflow on different case studies. By implementing this workflow in real-world deconstruction and construction scenarios, our contribution lies in developing effective strategies for scalable, globally applicable circular building practices. Table 1 summarises the digital technologies tested in our case studies to develop the workflow.

**Table 1:** Main digital technologies explored in the case studies for validating the D5 workflow

Digital technologies	Purpose	Steps
Machine learning (ML)	To conduct comprehensive assessments of existing building stocks from building records in combination with geographical information systems (GIS) and to assess the identified stocks to estimate the potential for reusing building components.	Detection
Computer vision (CV)	To advance material recognition from visual data and automate the disassembly-for-reuse process, identifying material types and conditions during deconstruction for precise classification.	Detection Disassembly
Reality capture	To generate 3D geometric data of existing materials, integrating this information with BIM systems as cyber-physical elements. In combination with robotics, these technologies enable systematic deconstruction and sorting processes to facilitate the careful disassembly of building materials.	Detection Disassembly
Extended reality (XR)	To aid in the disassembly and reassembly of materials, simplifying the process and ensuring accuracy in fitting reclaimed components. Robotics are also explored to disassemble building elements carefully.	Disassembly Deployment
Digital product passports (DPPs)	To extract data to feed into specialised algorithms tailored for the construction industry to effectively match the supply of available materials with demand, serving as digital intermediaries for stakeholders across the value chain.	Distribution
Track & trace technologies (including Internet of Things (IoT) & data carriers)	To track information on materials to connect DPPs and material databases.	Distribution
Decentralised storage technologies (e.g., blockchain)	To trace the history of the material and information providence.	Distribution
Generative AI	To enhance creativity in the architectural design process with reused materials.	Design
Computational design algorithms (e.g., parametric design)	To plan and model buildings specifically using reused materials. Algorithms are improved to accommodate existing material inventories while factoring in the variances necessary for working with reclaimed stock.	Design
Digital fabrication (e.g., additive and subtractive manufacturing)	To produce precise connectors that facilitate the integration of reused materials.	Disassembly Deployment

Case studies were used to validate the developed workflow for digital technologies in each circular construction step (Table 2). One case study is the City of Zurich, for which CV and ML were used to document building materials and predict material stocks. Another set of studies focused on the disassembly of a Geneva warehouse and a Zurich music pavilion, where automated material cataloguing and sorting technologies were tested to optimise the reuse process. In addition, full-scale applications involved the disassembly of these buildings, followed by the design and assembly of Dome 5.1 and Dome 5.2 with the materials reclaimed from the warehouse and music pavilion. Emerging technologies were further explored in the set of domes called Dome 5.x, exploring generative AI and XR to innovate design and improve structural assembly processes.

**Table 2:** Synthesised D5 Workflow from case studies and enabling technologies to upscale the reuse of building materials (as explained in “Methods”)

	1. Detection	2. Disassembly	3. Distribution	4. Design	5. Deployment
To fully upscale reuse from the existing building stock, we need to:	Identify buildings pre-demolition	Invent materials for post-disassembly sorting	Give materials an identity for new construction sites	Design with the available stock of reused materials	Fabricate buildings with bespoke reused materials
Methods	Data and image processing to identify material stock in cities	Geometry and material identification	Database management for tracking, tracing and trading	Human-machine interaction for creativity support	Connection fabrication and assembly
Digital technology	• ML • CV	• Reality capture • Scan-to-BIM • CV • XR	• DPPs • Tracking and tracing technologies • Blockchain	• Generative AI • Computational design algorithms	• Additive & subtractive manufacturing • XR
Case studies	• Zurich City	• Geneva warehouse • Zurich music pavilion • K118	• Database of disassembled timber beams for reuse in domes	• Dome 5.1 • Dome 5.2	• Dome 5.1 • Dome 5.2 • Dome 5.x
					

### D5 digital circular workflow

**Fig. 1:**
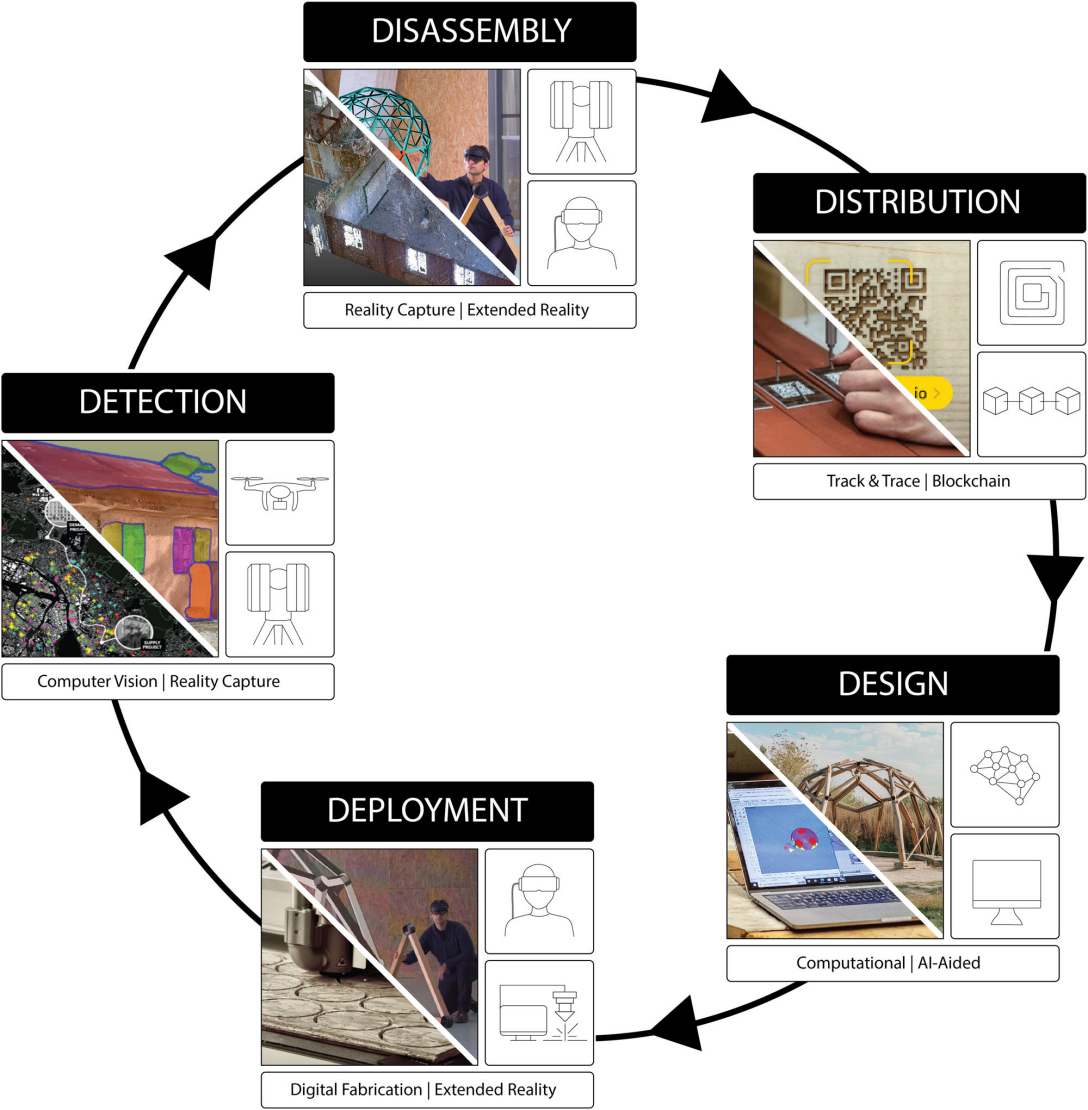
D5 Digital Circular Workflow (“D5 Workflow”).

Through the case studies in Table 2, we developed a five-step D5 Digital Circular Workflow (Fig. 1) to match the supply and demand of reused materials. Our research demonstrated that the collected material information could link to existing material marketplaces for broader distribution (in Switzerland in our cases). By developing the digital foundations for this matchmaking^85,86^, the research aims to contribute to the upscaling of circular economy principles within the built environment.

#### Step 1: Detection

Learnings from our analysis of the City of Zurich demonstrated how to use a data-centric approach to harness urban data sources, such as Google Street View, cadastral records, and diverse photography— indeed, before demolition, buildings should be classified as repositories of reusable materials.

CV and ML algorithms can identify and catalogue materials for reuse, supported by advanced scanning for 3D representations integrated into BIM. Cadastral data and public record (e.g., Open Data Zurich) enable the creation of building classification maps outlining material-specific characteristics^87^. Google Street View imagery, combined with CV and ML, is used to identify the existing material stock that needs to be dismantled or renovated. Such tasks are traditionally labour-intensive if pursued without automated methods. Therefore, algorithms are developed to enrich building databases, amalgamating data collected from images, public records and cadastres^88^. By establishing the composition and condition of materials in urban structures, resource optimisation strategies can be developed to minimise waste generation and foster circular economy practices.

**Fig. 2:**
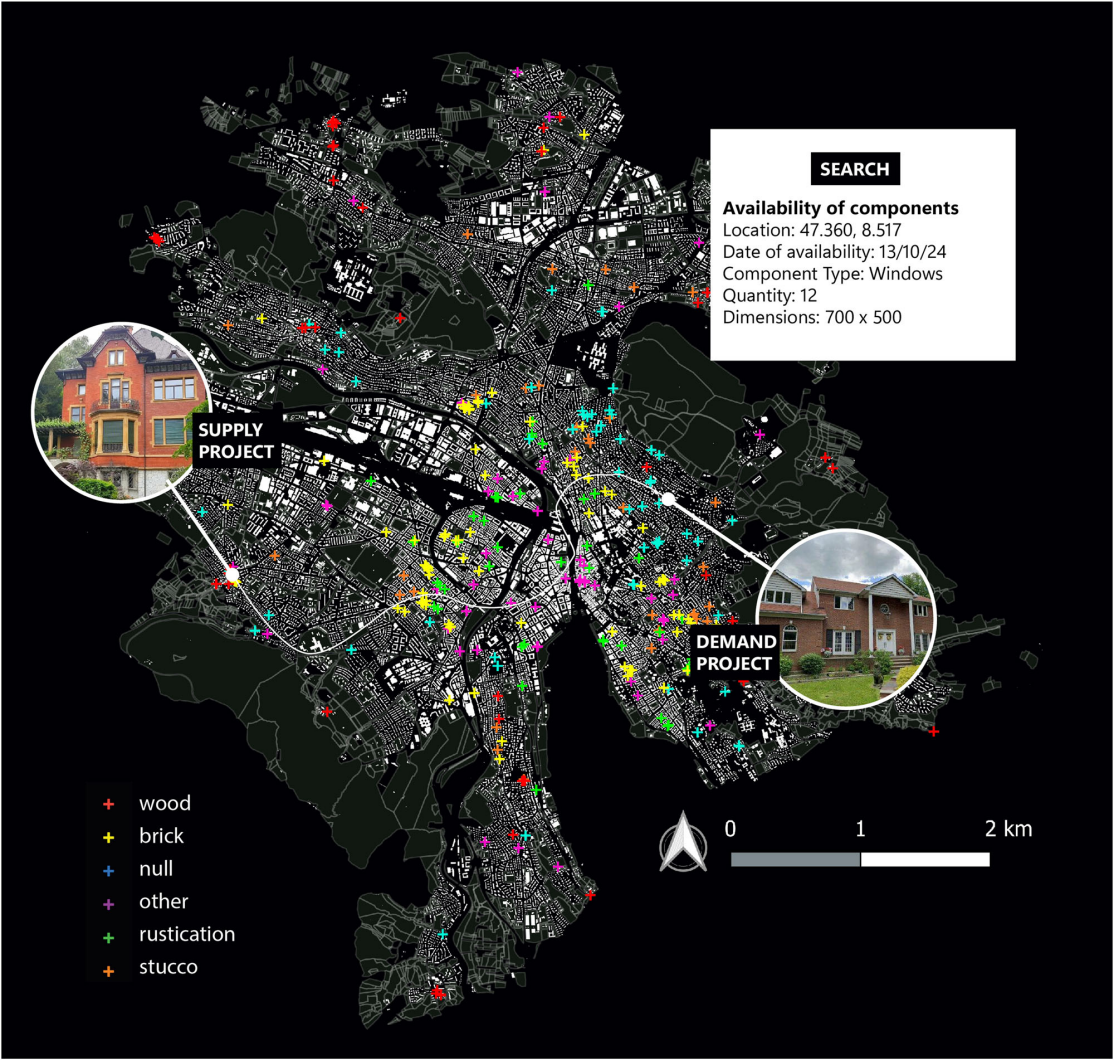
Spatial distribution of building materials for a subset of buildings in Zurich, Switzerland, adapted from ref. ^88^.

The detection method also incorporates real-time analysis of images or digital feeds from building owners, contractors, or even citizens, improving database accuracy and usefulness by incorporating a broader and more current range of materials and conditions. This enhanced spatiotemporal understanding of material concentrations (i.e., insights into where specific materials are most abundant) aids urban planners in pursuing a circular economy through strategized deconstruction or renovation efforts (Fig. 2).

#### Step 2: Disassembly

Learnings from disassembly sites in Geneva and Zurich, Switzerland, demonstrated how scan-to-BIM and CV tools lead to the effective cataloguing of materials into expansive databases.

Typically, only 1% of building materials are reclaimed in deconstruction projects^19^. The disassembly process involves on-site dismantling, sorting materials on- and off-site, and cataloguing materials in a database for further distribution. We explored how digital technologies can support systematic disassembly, making it safer, more affordable, more efficient and healthier than conventional demolition. The applied methods document geometries and material specifications to assist in disassembling materials marked for reuse. Integrating advanced CV, XR and robotics enables the automation of complex disassembly processes while incorporating human reasoning and adhering to industrial protocols.

**Fig. 3:**
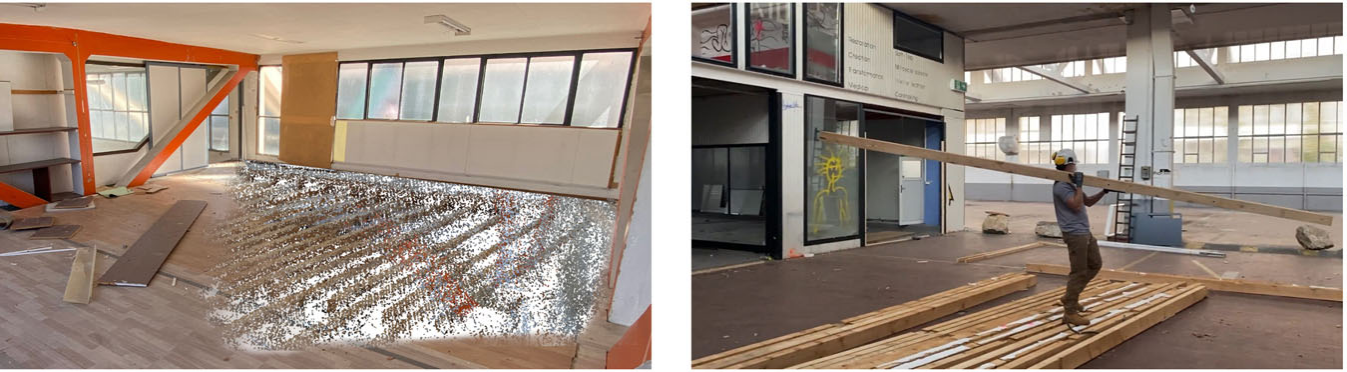
Geneva warehouse disassembly for building Dome 5.1. Point clouds (left) of the disassembled beams and actual disassembly (right) of these beams.

**Fig. 4:**
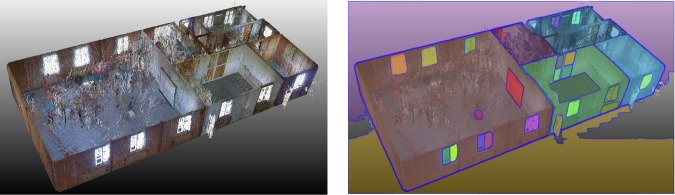
Zurich music pavilion and the cataloguing of materials that could be disassembled for the building of Dome 5.2. Raw point cloud generated through scanning (left) and segmented image processed through CV (right).

**Fig. 5:**
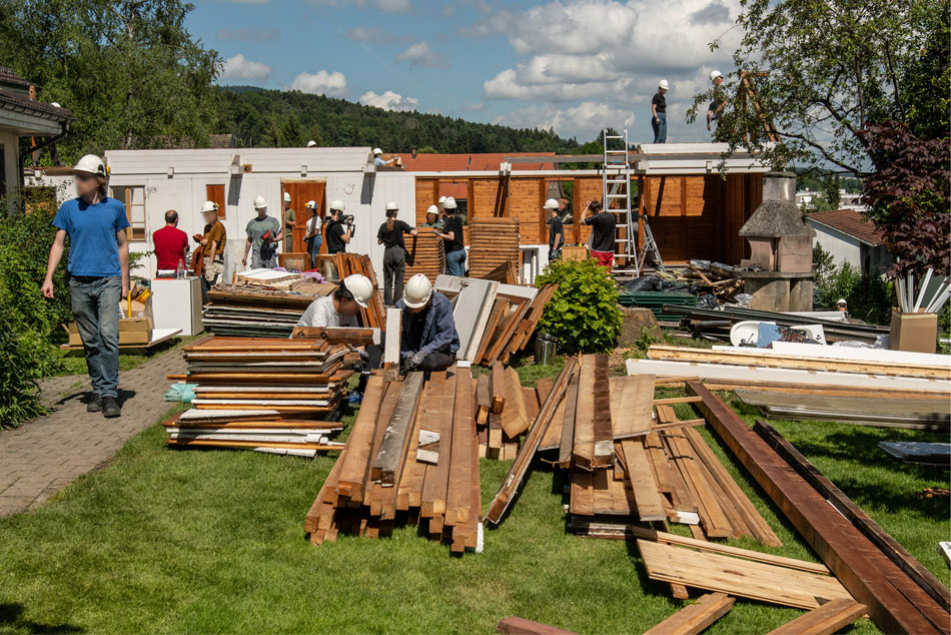
Disassembly of the Zurich music pavilion, after scanning and material identification, enabled the cataloguing and sorting of the materials, carried out by the authors in collaboration with industry practitioners and academic students (Photography: Buser Hill Photography).

Capture systems for building spatial data vary in technology and user interaction, and these include photogrammetry with software such as Agisoft Metashape or COLMAP and real-time geometry reconstruction tools such as RealityCapture. Various methods were used to capture imagery, such as helmet-mounted cameras for perspective and spherical capture (Fig. 3, left), smartphones (Fig. 4, left), drone-based systems for exterior scans, and LiDAR for high-accuracy measurements. These technologies enabled the pre-assessment of recoverable building components (e.g., ease of material removal estimation), using point density, CV, and graph-based matching. Using CV, we enhanced pre-demolition material recognition by detailing material types and conditions (Fig. 4, right). As images of these conditions are rare in bulk, training data was supplemented with industry images of materials rejected due to defects or damage. The disassembled building and sorting of materials in the Zurich case study is illustrated in Fig. 5.

Throughout the application of scan-to-BIM to identify steel beam and column systems in the K118 case study, photogrammetric methods outperformed mobile-phone LiDAR capture. There was also significant variation between the results for different software used to control the LiDAR capture.

#### Step 3: Distribution

Learnings from the distribution of the disassembled materials from the demolition sites in Geneva and Zurich, Switzerland, demonstrated how to conceptualise DPPs or digital identities for efficiently tracking, tracing, and trading building materials, facilitating the transition from demolition sites to new construction sites.

To upscale circular construction, it is important to understand the supply chain of building materials and their properties. We do this by labelling materials so they can be tracked through more accessible datasets. This enables us to match supply and demand of reused materials^35^—information is needed on who has ‘waste’ materials to offer, where they can be stored, and who wants to turn them into resources. However, rapid proliferation of passport-type mechanisms (building, material, or product passports) resulted in market confusion throughout the entire construction sector, as various platforms are collecting different levels of detail for different purposes (marketplace, circularity calculator, etc.)^89^.

To facilitate communication and collaboration between value chain actors, BIM can be used to match data from design, procurement, and construction for recording data in a DPP. This DPP then contains relevant information such as building geometry, material properties, and quantities of components. The case studies showed the need for standardisation of these DPPs. To reach a consensus on these passport mechanisms, we continue to collaborate with stakeholders and policymakers from the building industry^86^. Tagging technologies such as quick response (QR) codes and radio frequency identification (RFID) chips, using Internet of Things (IoT) to connect components to DPPs, enable this transparent material tracking and tracing^90^.

#### Step 4: Design

Learnings from designing domes with the reclaimed materials from the disassembly sites demonstrated how to use generative AI to stimulate creative building design with reclaimed component and activate computational design algorithms to match the available materials with new construction projects.

**Fig. 6:**
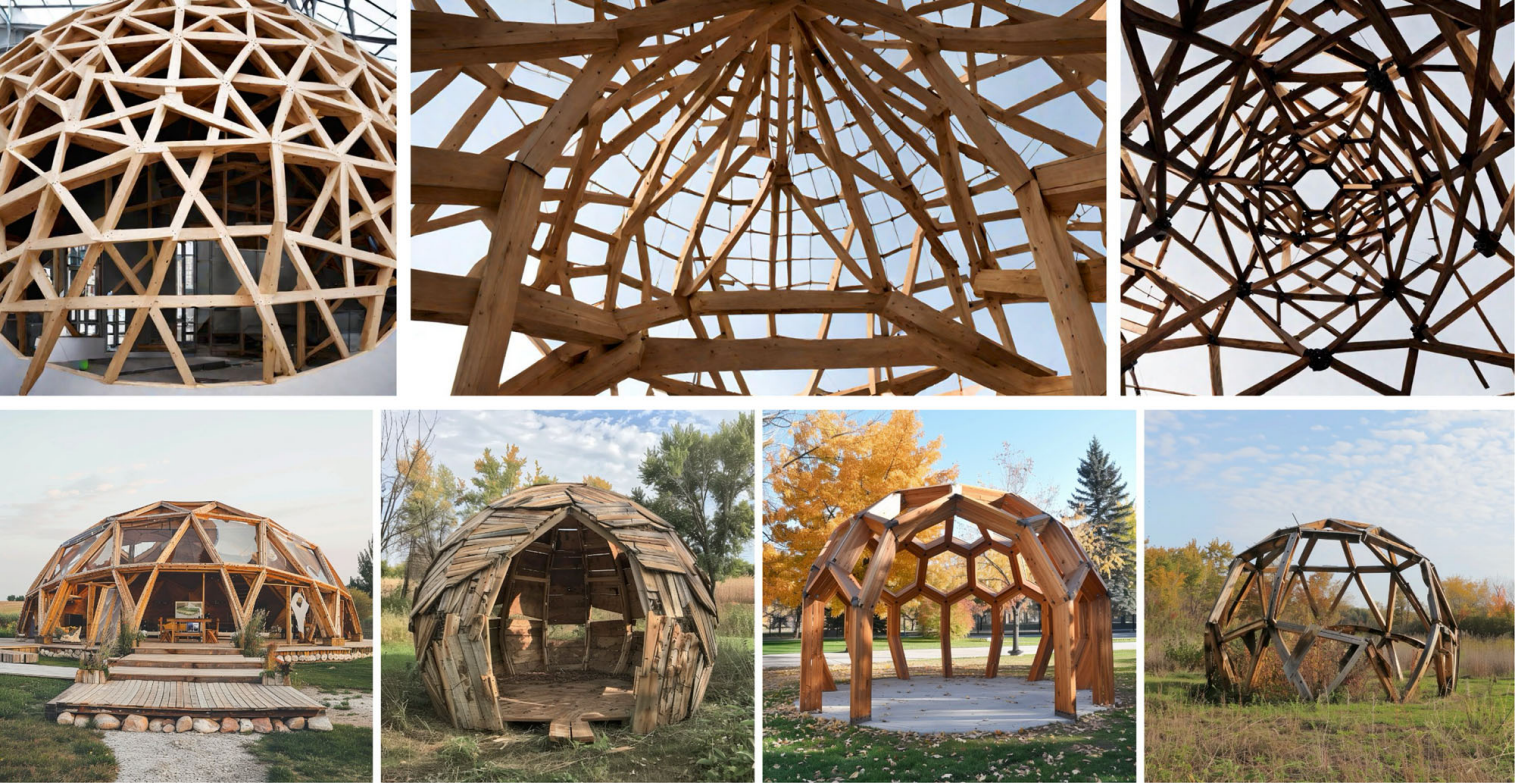
Images generated with RunwayML (top) and Midjourney (bottom) based on the text prompt: “Outside picture of a dome structure similar to Buckminster Fuller’s dome made out of repurposed wooden beams”.

Generative AI tools were applied in the early design stage of the dome case studies (Domes 5.x) in the form of Text-to-Image engines from RunwayML^91^ and Midjourney^92^ (Fig. 6). The aim is to explore if these tools can inspire architects to come up with creative design solutions for repurposed, often non-standardised materials. This approach helps incorporate repurposed materials into the design process by including them in text prompts. However, this method has limitations in generating practical solutions, as the outputs from the Text-to-Image models are two-dimensional (2D) images that do not represent structurally viable designs, nor do they respect the given material passports geometrically or physically. Despite these limitations, generative AI has potential for driving inspiration in future architectural design processes, particularly if integrated with 3D data to produce spatial outputs that can be more thoroughly evaluated. We also expect this field to rapidly evolve in the near future.

**Fig. 7:**
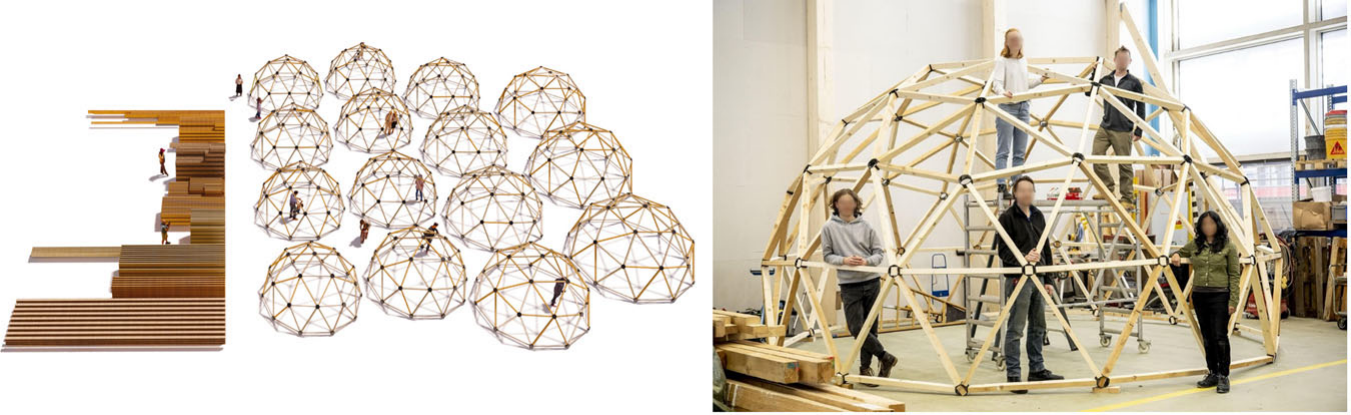
Dome 5.1 built with disassembled materials. Computational design^93^ (left) and test the construction (right) (Photography: Daniel Winkler).

Computational design enabled the more accurate design of the domes with reclaimed materials from our disassembled building case studies. Building upon a Grasshopper-based tool^93^, multiple geodesic domes can be created (see Fig. 7 Left), each with an adjustable radius and frequency, using wooden beams of various sizes. To apply this tool to our case studies (Domes 5.1 and 5.2), the matching strategy is adjusted from a one-to-one assignment problem to a one-to-many cutting-stock problem, using integer linear programming (ILP)^94,95^. A design optimisation algorithm adjusts parameters to maximise floor area and material usage while minimising waste. Cutting-stock optimisation then matches available stock with design needs. Reuse presents unique challenges, as minimising cutoff waste increases operator time due to frequent tool changes and non-sequential production. To address this, two additional goals are integrated into the ILP optimisation: reducing component variety from a single stock piece and sequencing production efficiently^96^. The design’s stability also accounted for uncertainties or errors in stock availability (Fig. 7).

**Fig. 8:**
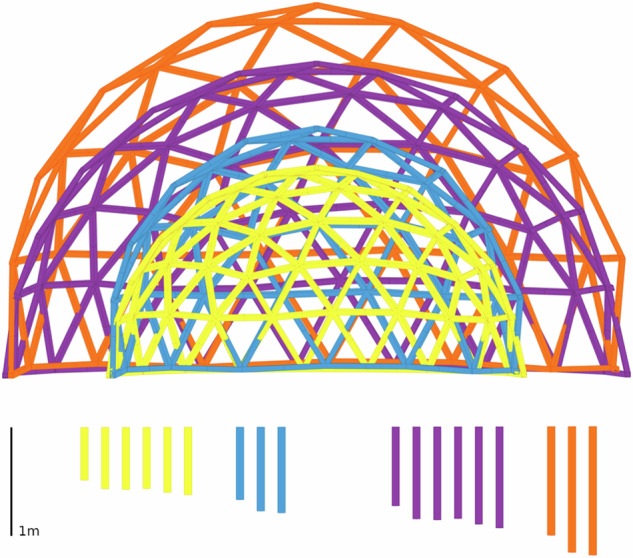
Computational design for circular construction of Domes 5.1 and 5.2: design extents of the dome, with typical parts for each case below.

With the ultimate goal of optimising material usage (Fig. 8), an objective to minimise waste was tested against one maximising the contiguous length of remaining pieces to potentially enhance the value of larger components. In simulations matching a test dome design requiring 170 m of material to various inventory scenarios, the waste-minimising objective generally resulted in a more favourable waste score, typically by 4 m or less, but up to 12 m as the available inventory increased. However, the objective focusing solely on contiguous lengths produced scores that were only marginally better, within 1% of those achieved by the waste objective and both within 10% of the theoretical maximum, suggesting that it might be more suitable as a secondary factor. New objectives focusing on production order and fabrication tooling changes were also compared. An objective centred on production order generally resulted in waste scores that were 15 m worse than those achieved by the waste-minimising objective, and it did not surpass 95% of the theoretical maximum contiguous score. Similarly, the tool-change objective led to waste scores that were 20 m or more and did not exceed 90% of the theoretical maximum contiguous score. These findings suggest that while these objectives may not be as suitable for smaller-scale constructions like the dome, they could be more beneficial in larger projects with more complex scheduling requirements.

#### Step 5: Deployment

Learnings from assembling the designed domes demonstrated how subtractive and additive manufacturing facilitate the making of connections between bespoke reclaimed elements and how innovative tools such as XR can facilitate the assembly of the refabricated components with reclaimed materials in new construction projects.

**Fig. 9:**
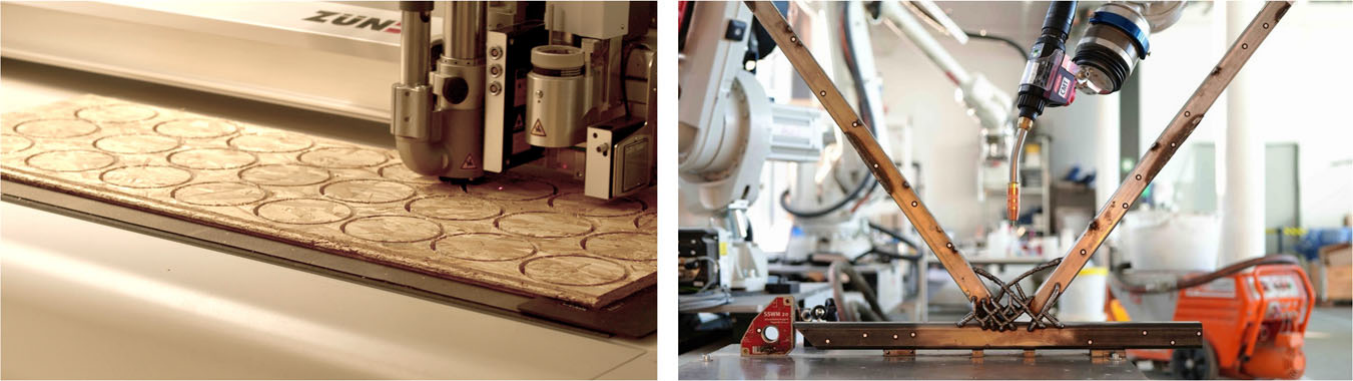
Digital fabrication for circular construction. Subtractive manufacturing of dome connectors (Circular Engineering for Architecture, Photography: Alexander Attias) (left) and adaptive detailing with robotic additive joining for reclaimed steel^97^ (Gramazio Kohler Research, Circular Engineering for Architecture and Chair of Steel and Composite Structures, Photography: Inés Ariza) (right).

The integration of additive and subtractive manufacturing techniques plays a pivotal role in the adaptation and reuse of materials, thereby contributing to more efficient circular supply chains. Specifically, computer numerical control (CNC) milling—a form of subtractive manufacturing—was employed to create bespoke connectors from oriented strand board (OSB) plates (which were about to be thrown to waste from the Geneva disassembly site) in the Domes 5.1 and 5.2 case studies. These connectors were designed to enhance the structural integrity of the reclaimed water pipes. Indeed, reused materials are not always the exact dimensions needed for the new construction and need to be augmented to meet the required dimensions. This customisation capability afforded by digital fabrication methods proves essential in accommodating the non-standard sizes frequently encountered in reclaimed materials (Fig. 9, left). Furthermore, additive manufacturing enabled robotic additive joining—a form of additive manufacturing—with steel to produce unique connections for reused steel beams^97^ (Fig. 9, right). This approach not only demonstrates the feasibility of tailored component interfaces in construction but also underscores the potential of digital fabrication technologies to foster material reuse and reduce waste in building environments.

**Fig. 10:**
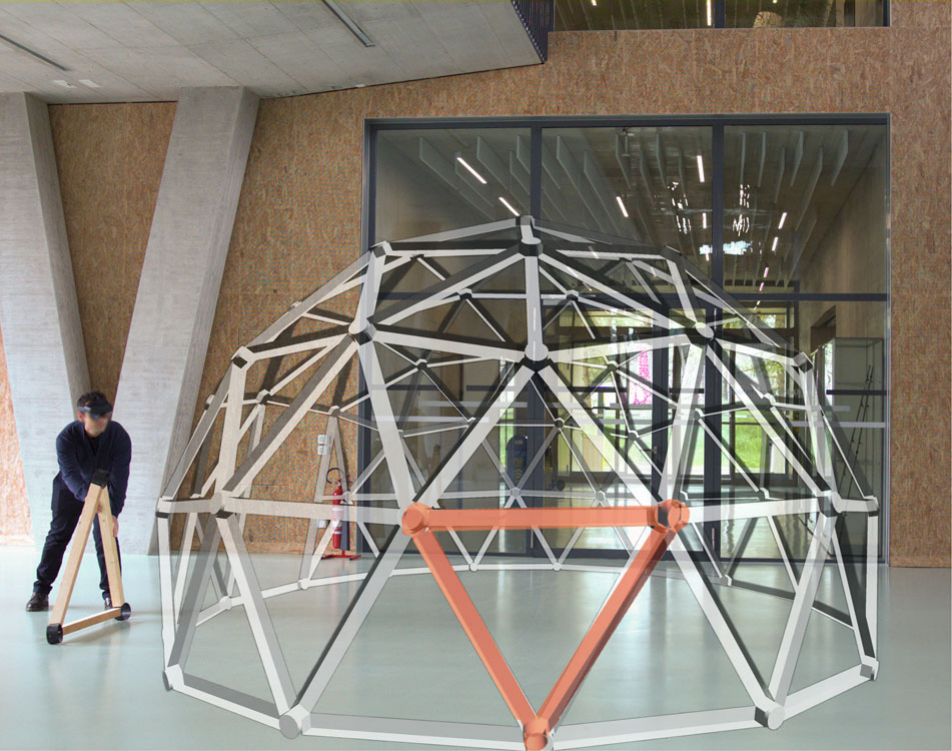
Using XR for the structural assembly of a dome with reclaimed elements.

Using a state-of-the-art XR head-mounted display (HMD), in this instance the Microsoft Hololens 2, enabled a step-by-step guided assembly of a dome with reused materials (Fig. 10). The simulation engine Unity3D was used to facilitate the visualisation of the 3D dome based on an accurate 1:1 3D model of the structure. The 3D dome was projected on the HMD, with a slight transparency to it, within a physical space located at the facilities of ETH Zurich. Using different colour schemes directly projected to the HMD (such as the orange in Fig. 10) can provide valuable spatiotemporal information, which is critical in engaging and immersing the user as intuitively as possible, especially when incorporating XR technologies^82^. From a practical perspective, this colour-coding system guides the user in the assembly process in identifying which exact component needs to be assembled and in what particular order across the diverse components (temporal information) as well as in determining where exactly the component needs to be placed and mounted (spatial information)^84^.

## Discussion

**Fig. 11:**
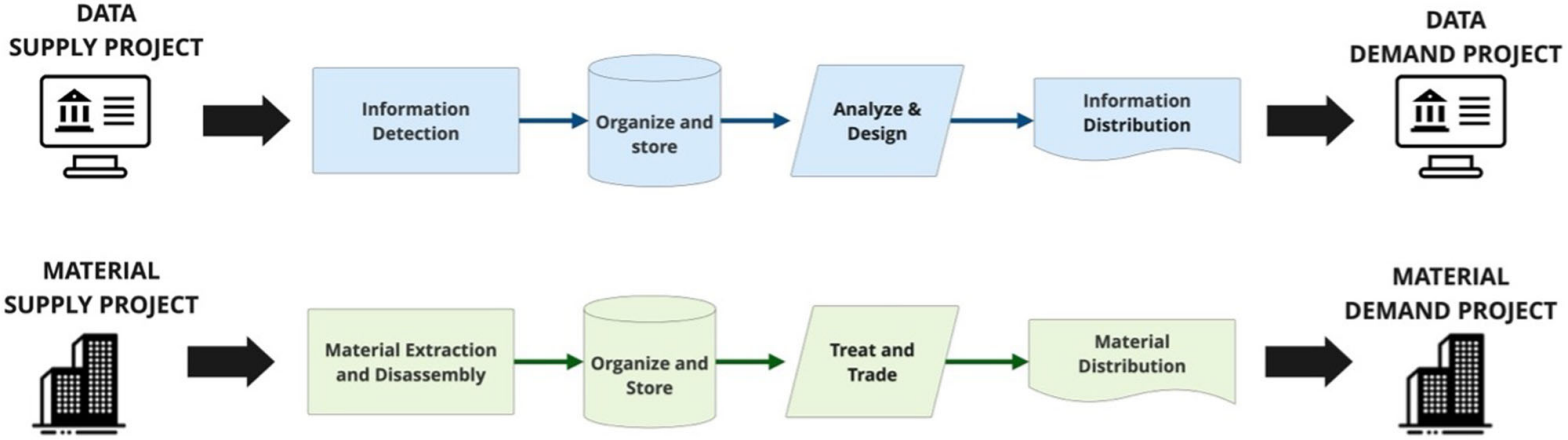
Data and material flow management for circular building projects.

Our study confirms difficulties in coordinating data among stakeholders due to a lack of information as a major obstacle to implementing circular supply chains and material reuse^98–100^. To address this, our findings suggest that improving the quality of shared data is critical. Aligning information and material flows beyond the initial phases of product and construction life cycles is rare, due to the complexities of managing and owning information in construction projects and the fragmented nature of the industry^101^. A structured approach to information flow is needed to identify key steps for reusing materials. Figure 11 illustrates such a process, showing the transition from the end-of-life of one building project to the start of another project, highlighting the similarities between the digital and physical processes for construction reuse. The use of digital technologies in our proposed workflow enables the alignment of both data and material supply chain.

For the **detection** of materials available for reuse, accurate material classification for reuse in building facades has been achieved using CV and ML on street-level images and demolition datasets. Temporal data integration can enhance real-world applicability, necessitating continuous updates to capture renovation-induced changes. Addressing visual and cadastral data sparsity via data augmentation is critical for model scalability and reliability and to avoid biases. Furthermore, comprehensive building inventory analysis must extend beyond surface visuals to include underlying structural and material complexities. This involves advancing the current state-of-the-art in CV algorithms, using SVM-based methods tailored for the AEC sector and CNN-based methods known for their accuracy in general vision tasks in combination with detailed on-site material and structural analysis. The approach could integrate specific data through an extension of our process to generate detailed depth maps, BIM-type mappings, and non-photographic sensor data to enrich the overall dataset.

For the **disassembly** of buildings, we validated the development of material geometry analysis to catalogue the materials through the case studies. Previous research primarily provided either general spatial overviews (such as rooms and floor plans) or detailed analyses of specific features (such as space frame node positions)^102^. Methods for analysing beam and column components were tailored to accommodate the anticipated geometry range, ensuring accurate data extraction^103^. Error correction methods were tailored for different components, addressing significant discrepancies from incorrect beam splits or joins that affected size estimation. Though our focus was on dimensional accuracy, no standards define what constitutes a ‘usable’ reconstruction. As CV for material recognition and inventory in disassembly advances, future research should improve these techniques’ accuracy and robustness for better material identification and classification under complex conditions. Real-time visual data analysis can aid immediate decision-making, and specialised datasets and annotations can enhance model training. Combining CV with robotic systems and ML algorithms^104^ could automate disassembly and optimise material sorting. Integrating these technologies with environmental impact assessment tools would provide detailed analyses of material reuse benefits. Photogrammetry is approaching LiDAR in accuracy, with advancements like NeRF^105^ improving photo-based analysis. A hybrid approach, dividing sites for optimal method use, could balance capture time and accuracy. Future studies should expand digitisation sources, incorporating non-geometric sensors like thermal cameras, testing mobile-phone LiDAR, and developing a matchmaking database combining data from photogrammetry, LiDAR, sensing, and CV. This would support architects in designing innovative buildings based on available stock.

For **distribution**, material tracking and tracing in building construction have been streamlined through the use of QR codes linked to unique DPPs. These passports provide detailed histories and potential applications of building materials, information derived from data collected during the monitoring phase. This system has the potential to transform the industry by creating a dynamic marketplace for the redistribution of materials from deconstructed buildings, effectively matching supply with demand. Furthermore, it could integrate with broader material marketplaces, establishing a more extensive and sustainable network for material reuse across the construction industry. This approach not only facilitates efficient material management but also contributes to the development of more sustainable construction practices. The construction industry’s fragmented supply chain presents challenges in material traceability. Blockchain technology, a secure distributed peer-to-peer system, offers a potential solution for transparent value transactions without the need for central authorities and intermediaries^58^. Part of the challenges associated with reusing elements is the lack of information availability and distribution, both digital and physical. Data from physically monitoring components allows for an understanding of how properties change over time, which could inform its future use case. The subsequent question is how they are being monitored and where that information is being stored. Future data distribution can leverage linked data principles, distributed ledger technologies, cloud computing, decentralised identity and storage, and open data platforms.

For the **design** step, AI-driven designs, while currently showing limited constructability, have significantly advanced traditional architectural thinking and practice. While AI could generate numerous innovative design ideas, most demonstrated limited practical use in real construction. However, these outcomes proved invaluable in demonstrating the potential of AI to inspire creative thought processes among architects. The designs, although not always directly usable, sparked new ideas and discussions about the possibilities of material reuse, thus contributing significantly to the conceptual phase of the architectural design of future domes. This underscores the need for continual refinement of AI models to better meet practical construction requirements, merging human oversight with AI’s innovative capabilities for a balance between creativity and functionality. Using 3D data to train machines and produce outputs holds the potential to further integrate AI in circular design strategies. The connection of optimisation tools with design tools at the initial stages of design development and adapting design mindsets to broader definitions of optimisation are essential. This involves considering factors like future usability and production processes of components. In addition, the design system’s approach to changes—as mere material reassignments—overlooks significant acceptance and aesthetic impacts, highlighting an area for improvement in optimisation strategies. Design optimisation scenarios should cover more realistic conditions, especially multi-source to multi-design over single-source to single-design, to further assess the effects of variable transport distances, component conditions, and manufacturers. While the problems of 1D and 2D material cutting and matching are well studied, a more generalisable strategy should be developed for assessing the matchmaking of arbitrary components. Moreover, the large difference in objective results and timing between goals in the computational design optimisation of the Domes 5.1 and 5.2 indicated the need to precisely define the production needs of a particular design. While goals focusing first on cutoff waste will consistently produce the best results for this one factor, this is unlikely to be the only consideration in real-world scenarios.

The **deployment** of buildings with reused materials can be transformed by recent advancements in XR technologies, AI, robotics, and digital fabrication research in construction. These innovations improve the efficiency and effectiveness of complex assembly processes in the AEC industry, particularly in reassembly and disassembly, as showcased in the dome case studies. However, further research is necessary to quantify the benefits of XR technologies and develop a multifaceted evaluation strategy with diverse spatiotemporal metrics. This will provide a deeper understanding of XR’s value and allow comparison with traditional methods. Understanding how XR enhances assembly and human motor performance could lead to interfaces that augment user capabilities and automate labour-intensive tasks, freeing engineers and architects to focus on high-level decision-making^84,104^. Integrating multi-sensory feedback (sound and tactile) in the assembly of reused materials can enhance performance by reducing reliance on visual cues and leveraging human sensorimotor strengths^82,83,106^. By incorporating XR technologies with robotics, enhanced sensory feedback allows users to intuitively interact with their surroundings, enabling robots to automate complex tasks through advanced AI methods^84^. Such integration of XR, AI, and robotics facilitates the replication of complex human behaviours and skills, optimising the automation of demanding, lengthy, and arduous tasks in robotic manipulation and assembly^104,107^.

In conclusion, this paper presents a unique approach that harnesses digital innovations from other sectors to enhance the skills of stakeholders in the AEC sector through integration from the broader field of computer science and in the narrower context of human-computer interaction. By leveraging these digital tools, our research aims to disrupt the current linear value chain of the construction sector and establish a data-driven digital circular design and construction approach that promotes effective, user-friendly, and widespread reuse of building materials. The learnings from the case studies not only contribute to the advancement of a sustainable architecture workflow yet also showcase the power of interdisciplinary collaboration. The D5 Workflow is essential in achieving the ambitious goal of zero-carbon buildings by 2050. Furthermore, our hands-on, project-based learning approach demonstrates that it is an effective way to acquire circular engineering and design skills in a real-world design and construction project. Future research aims to expand the workflow into a matchmaking service^85,86^ that pairs supply (materials available for reuse, skills, tools, etc.) and demand (builders in need of materials, skills, tools, etc.) through cloud computing, blockchain, automation, robotics, and big data analytics.

## Methods

We adopted an action research-based methodology to constructively bridge the gap between theoretical insights and practical applications of circular economy principles in the AEC sector^108^. Following problem identification, we developed an action plan for digitising circular construction processes, called the D5 Workflow, and evaluated the learnings in each of the five steps. Subsequently, these steps were discretely validated through multiple case studies. To apply this circular model in the current and future AEC sector, we worked closely together with construction industry practitioners to exchange and disseminate knowledge^108^. The case studies are described in Table 3 and the integrated learnings from each implemented action are synthesised in “Results”.

**Table 3:** Case studies using the D5 Workflow and references of each case study

Case study	Description	Step	References
Zurich City	GIS data, cadastral data, demolition audit data, and Google Street View data were collected	Detection	^43,87^
Geneva Warehouse	A steel and timber floor structure were disassembled pre-demolition. The timber beams, pipes, and OSB plates were reused for Dome 5.1.	Disassembly	^103^
Zurich Music Pavilion	An entire two-floor timber building was disassembled. The timber beams were reused for Dome 5.2.	Disassembly	^96^
K118	A reused steel structure and interior of the building was scanned, making a material inventory.	Disassembly	^110,113^
Dome 5.1	A first dome was built with the reused elements from the Geneva warehouse. The dome has been disassembled and re-assembled at four different locations. QR codes were engraved for the material passport tracking. CNC milling was used for the connection fabrication.	Distribution Design Deployment	^90^
Dome 5.2	A second dome was built, improving the tracking technologies, design modelling, and fabrication technologies.	Distribution Design Deployment	^55^
Domes 5.x	Generative AI and XR workflows were tested out on building new domes with reused materials.	Design Deployment	^84^

### Urban-scale case study: Zurich City

The City of Zurich was chosen as a case study to investigate building types and material patterns to enhance circular construction at an urban scale. A notable method involves using publicly accessible street-level imagery combined with CV, ML and GIS data to document building facade materials, addressing the challenge of limited data on existing materials. This approach uses non-proprietary data, making the technology accessible for broad implementation^108^.

Further research derived from integrating open-access cadastre data with semi-open demolition audit records also examined the documentation of material information from existing buildings^43^. It employs three predictive ML algorithms: linear regression, random forest regressor, and extreme gradient boosting on 409 residential buildings in Zurich. This model predicts the quantities of various materials like wood, minerals, metals, glass, and roof tiles in existing buildings.

For this study, cadastral information from Open Data repositories in Zurich was queried to examine the spatial location of building types: residential, commercial and industrial. This mapping helped identify the distribution and potential for material reuse among different building structures. In addition, the buildings’ year of construction was overlayed to identify locations of buildings that may require circular retrofitting or repair due to their age. Areas in which new construction is contracted were also examined to locate potential sites of building disassembly or deconstruction. Street view images of the buildings were then retrieved using Google’s API to visually examine the building architectural style and exterior materials. By integrating spatial analysis with images and other construction data, the study provides a method to identify targeted opportunities for material reuse in an effort to advance circular construction practices.

### Disassembly case studies: Geneva warehouse and Zurich music pavilion

A car warehouse in Geneva, Switzerland, and a music pavilion of a hospital in Zurich, Switzerland, were carefully disassembled by the authors to test the automation of material cataloguing and sorting using reality capture technologies.

First, physical digitisation methods were evaluated for use in initial assessments of buildings about to be demolished. Each method explicitly or implicitly produces geometric information in the form of point clouds, coloured 2D imagery, and a spatial record of the locations of capture in the site. Capture methods were first evaluated for their feasibility in terms of accuracy and efficiency at the building scale^47^.

Available capture systems vary by both their underlying technology as well as the specifics of user interaction. First, photogrammetry reconstructs spatial data based on dense collections of 2D site photography. The relevant software may be an all-in-one product, such as Agisoft Metashape, or a modular pipeline such as COLMAP that can be further integrated with other analysis tools. In addition, software may operate off-site after data collection, or reconstruct live geometry as successive photos are taken (e.g., RealityCapture)^109^.

To investigate their density of data and ease of use, several methods of imagery capture were compared, including perspective capture using a smartphone and spherical capture using a helmet-mounted camera, capturing both individual frames and video. These methods of human-guided image capture, appropriate for interior and detail work, differ significantly from drone-based capture, which is appropriate for larger areas and facades but more difficult for interiors. Next, we tested the efficiency and accuracy of LiDAR-based sensor systems. This covered the application of smartphone-based and handheld models with greater operator control, and tripod models with higher density and accuracy. The capture methods were compared based on their capture time, point density, overall accuracy, and applicability for simple geometric analysis. LiDAR methods were also evaluated for their use in material volume estimation^110^.

**Fig. 12:**
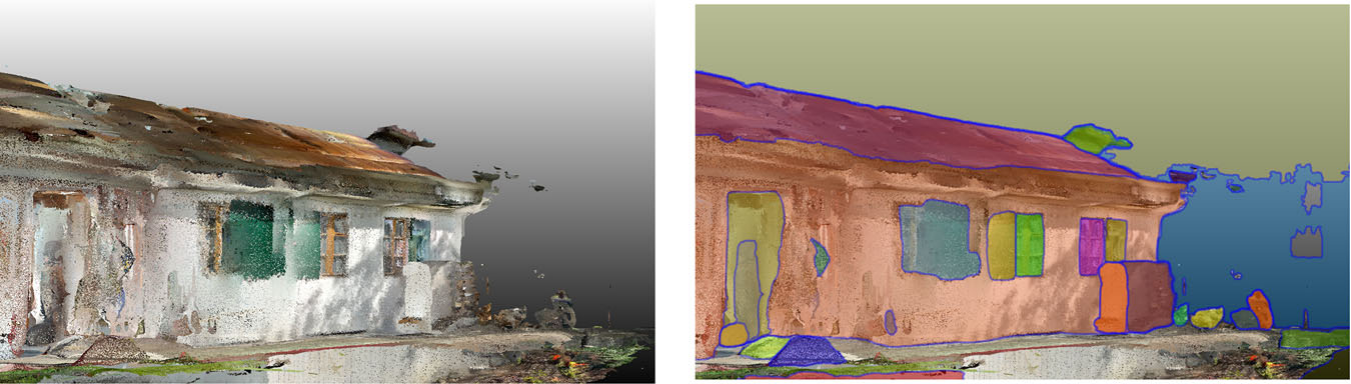
CV-assisted segmentation of an image of a scanned point cloud at Zurich music pavilion, showcasing digital material cataloguing techniques for optimised disassembly planning.

Scan-to-BIM methods were compared in terms of accurate element counts, major dimensions, and element relationships, given that the intended goal of the project was to pre-assess recoverable components from a space before disassembly. Methods based on point-density statistics and computer-vision detection (Fig. 12) were used to determine the location and bounds of elements for disassembly. Individual adjacent elements were progressively connected to the BIM model to represent the system as a graph, assigning each element an ease-of-removal score based on how many elements depend on it^96,111^. A stack-based model using both a bag-of-visual-words and CNN classifier achieved a 0.78 accuracy in classifying interior materials under varying conditions, with the CNN submodel consuming most of the training time^112^.

### Reuse building case study: K118

**Fig. 13:**
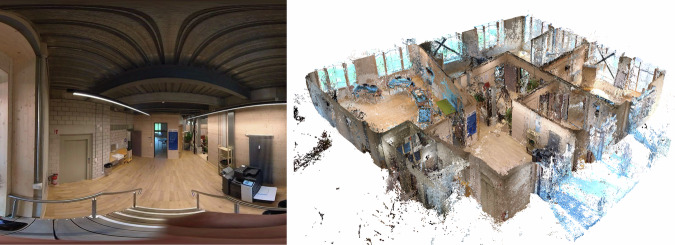
Representative photogrammetry input image and resulting cloud for case study building K118.

As part of the development of reality capture technologies for the detection and disassembly steps, the K118 Halle^21,110,113^, a building famous for its reused materials in Switzerland, has been used as a case study. The quality of scan sources was measured on factors including the density by surface area, degree of statistically detected noise, average deviation from a ground truth BIM model, and degree of surface coverage compared to a model (examples of this method tested on different buildings are shown in Fig. 13). The noise profiles of each capture method also affected component classification and localisation.

### Full workflow case studies: Dome 5.1 and Dome 5.2

With the materials disassembled from the disassembly sites (see above), two domes were built on the campus, then disassembled, distributed, and re-assembled. This enabled the full-scale application of our workflow. Tracking and tracing technologies were explored for the Dome 5.1 and Dome 5.2 case studies^55^. In the monitoring data phase of the projects, data collected from building audits (primarily through the visual audits mentioned above), deconstruction, and reprocessing were compiled in DPPs that were used to create new designs.

Next, to manage the data, materials had to be labelled to enable tracking and tracing. To ensure the continuity of information over several life cycles, approaches were developed to connect the web-stored DPPs to the physical building elements. The case studies used QR codes to simplify data access. Now, the material data collected during monitoring can be viewed by scanning the QR codes on the components.

Tracking and tracing enable us to store all information in one extendable database that can then be used to design new structures with reused materials. The amount of new cutoff waste was used as the objective value for the one-to-many stock-cutting problem (Fig. 8).

### Further emerging technology case studies: Domes 5.x

The design, disassembly and reassembly of the domes have been explored further (Domes 5.x), to evaluate rapidly evolving technologies such as generative AI and XR, as well as to test the ease of reuse of the materials from the original demolition sites throughout several different life cycles. This research used generative AI tools, specifically text-to-image engines from RunwayML^91^ and Midjourney^92^, in the early design stage of new dome projects to inspire architects with creative designs for repurposed materials, although the generated 2D images were not structurally practical or material-specific.

Moreover, XR technologies were employed for the case study as a proof of concept for the structural assembly of a timber dome on campus, composed of the reused materials from previously disassembled dome structures (Fig. 10). XR offers real-time, step-by-step visual guidance that transcends the spatial and temporal limitations of conventional methods. This case study highlights XR’s potential in enhancing assembly processes, with promising prospects for integrating XR with robotics to automate complex tasks through advanced AI, thereby improving efficiency and reducing manual labour. Future research on the intersection between XR technologies and robotics is being explored on these Domes 5.x.

## Data Availability

No datasets were generated or analysed during the current study.

## References

[1] United Nations Environment Programme. Building Materials and the Climate: Constructing a New Future. Nairobi. https://wedocs.unep.org/20.500.11822/43293 (2023).

[2] European Commission. Proposal for a Regulation of the European Parliament and the Council establishing a framework for setting ecodesign requirements for sustainable products and repealing Directive 2009/125/EC. https://eur-lex.europa.eu/legal-content/EN/TXT/?uri=COM%3A2022%3A0142%3AFIN (2022).

[3] Stahel, W. R. The circular economy. *Nature* **531**, 435–438 (2016). 27008952 10.1038/531435a

[4] Brand, S. *How Buildings Learn: What Happens After They’re Built?* (Penguine Education, 1995).

[5] McDonough, W. & Braungart, M. *Cradle to Cradle: Remaking the Way We Make Things* (North Point Press, 2010).

[6] Hebel, D., Wisniewska, M. H. & Heisel, F. *Building from Waste: Recovered Materials in Architecture and Construction* (Birkhaüser, 2014).

[7] Ellen McArthur Foundation. Towards the circular economy. www.ellenmacarthurfoundation.org/business/reports (2019).

[8] Lee, H. & Romero, J. Climate change 2023: synthesis report. Contribution of Working Groups I, II and III to the Sixth Assessment Report of the Intergovernmental Panel on Climate Change. Intergovernmental Panel on Climate Change, Geneva, Switzerland, 10.59327/IPCC/AR6-9789291691647 (2023).

[9] United Nations Environment Programme. The Buildings Breakthrough: Global push for near-zero emission and resilient buildings by 2030 unveiled at COP28. https://www.unep.org/news-and-stories/press-release/buildings-breakthrough-global-push-near-zero-emission-and-resilient (2023).

[10] CE100. Circularity in the built environment: case studies. A compilation of case studies from the CE100. (Ellen McArthur Foundation, 2018).

[11] Densley Tingley, D., Giesekam, J. & Cooper-Searle, S. Applying circular economic principles to reduce embodied carbon. In *Embodied Carbon in Buildings. Measurement, Management and Mitigation* (eds Pomponi, F. et al.) 265–286 (Springer, 2018).

[12] Guerra, B. C. et al. Circular economy applications in the construction industry: a global scan of trends and opportunities. *J. Clean. Prod.* **324**, 129125 (2021).

[13] Iacovidou, E. & Purnell, P. Mining the physical infrastructure: opportunities, barriers and interventions in promoting structural components reuse. *Sci. Total Environ.* **557–558**, 791–807 (2016). 27054305 10.1016/j.scitotenv.2016.03.098

[14] Chopin, J. & Delon, N. *Matières Grises* (Editions du Pavillon de l’Arsenal, 2014).

[15] Gorgolewski, M. *Resource Salvation, The Architecture of Reuse* (Wiley Blackwell, 2018).

[16] Addis, B. *Building with Reclaimed Components and Materials. A Design Handbook for Reuse and Recycling* (Earthscan, London and UK, 2006).

[17] Cheshire, D. *Building Revolutions, Applying the Circular Economy to the Built Environment* (RIBA Publishing, 2016).

[18] Superti, V., Houmani, C. & Binder, C. R. A systemic framework to categorize Circular Economy interventions: an application to the construction and demolition sector. *Resour. Conserv. Recycl.* **173**, 105711 (2021).

[19] Ghyoot, M., de Vlieger, L., Billiet, L. & Warnier, A. *Déconstruction et réemploi, comment faire circuler les éléments de construction* (Presses polytechniques et universitaires romandes, 2018).

[20] Stricker, E. et al. *Bauteile Wiederverwenden. Ein Kompendium zum zirkulären Bauen* (Park Books, 2021).

[21] De Wolf, C., Hoxha, E. & Fivet, C. Comparison of environmental assessment methods when reusing building components: a case study. *Sustain. Cities Soc.* **61**, 102322 (2020).

[22] Heisel, F. & Rau-Oberhuber, S. Calculation and evaluation of circularity indicators for the built environment using the case studies of UMAR and Madaster. *J. Clean. Prod.* **243**, 118482 (2020).

[23] Madaster: the cadastre for materials and products. Madaster Global. https://madaster.com/ (2024).

[24] Sumami. https://sumami.ch/ (2024).

[25] Materiuum. Rien ne se perd, tout se transforme! https://materiuum.ch/ (2024).

[26] Salza. Für die Wiederverwendung von Bauteilen. https://thetours.ch/ (2024).

[27] Zirkular. https://zirkular.net/en/ (2024).

[28] Syphon AG. Soziale Integration Biel und Seeland. https://syphon.ch/ (2024).

[29] Cirkla. https://cirkla.ch/en/ (2024).

[30] Chan, P., De Wolf, C. & Koutamanis, A. The digital potential in creating a circular construction economy. *Essay Counc. Environ. Infrastruct. RLI* https://www.rli.nl/sites/default/files/essay_3_the_digital_potential_in_creating_a_circular_construction_economy_-_tu_delft_paul_chan_def_1.pdf (2021).

[31] Ribeirinho, M. J., Mischke, J., Strube, G., Sjödin, E., Blanco, J. L., Palter, R., Biörck, J., Rockhill, D., & Andersson, T. McKinsey & Company. The next normal in construction: how disruption is reshaping the world’s largest ecosystem. https://www.mckinsey.com/~/media/McKinsey/Industries/Capital%20Projects%20and%20Infrastructure/Our%20Insights/The%20next%20normal%20in%20construction/The-next-normal-in-construction.pdf (2020).

[32] Çetin, S., De Wolf, C. & Bocken, N. Circular digital built environment: an emerging framework. *Sustainability* **13**, 6348 10.3390/su13116348 (2021).

[33] Yu, B. et al. Framework for sustainable building design and construction using off-cut wood. *NPJ Mater. Sustain.* **1**, 2 (2023).

[34] De Wolf, C., Çetin, S. & Bocken, N. (eds). *A Circular Built Environment in the Digital Age*. In *Circular Economy and Sustainability*. (Springer International Publishing, 2024).

[35] De Wolf, C., Cetin, S., Bocken, N. (2024). Can Digital Matchmaking Boost Circular Construction? Lessons from Reusing the Glass of Centre Pompidou. In: Thomsen, M. R., Ratti, C., Tamke, M. (eds) *Design for Rethinking Resources. UIA 2023.* Sustainable Development Goals Series. Springer, Cham. 10.1007/978-3-031-36554-6_42

[36] Zaadnoordijk, L., Besold, T. R. & Cusack, R. Lessons from infant learning for unsupervised machine learning. *Nat. Mach. Intell.* **4**, 510–520 (2022).

[37] Saxe, A., Nelli, S. & Summerfield, C. If deep learning is the answer, what is the question? *Nat. Rev. Neurosci.* **22**, 55–67 (2021). 33199854 10.1038/s41583-020-00395-8

[38] LeCun, Y., Bengio, Y. & Hinton, G. Deep learning. *Nature* **521**, 436–444 (2015). 26017442 10.1038/nature14539

[39] Akanbi, L. A., Oyedele, A. O., Oyedele, L. O. & Salami, R. O. Deep learning model for Demolition Waste Prediction in a circular economy. *J. Clean. Prod.* **274**, 122843 (2020).

[40] Spotr.ai. Inspect millions of buildings in seconds. https://www.spotr.ai/ (2024).

[41] Aeroscan. Kwaliteit en efficiëntie van vastgoed inspecties verbeteren? https://www.aeroscan.nl/ (2024).

[42] Kobyshev, N. *Towards Fully Automated City-scale 3D Reconstruction and Understanding*. Doctoral thesis, ETH Zurich (2017).

[43] Kobylinska, N. E., Raghu, D., Gordon, M., Hunhevicz, J. & De Wolf, C. Predicting recoverable material stock in buildings: using machine learning with demolition audit data as a case study. In *Presented at the EC3 Conference 2023, in Computing in Construction*, Vol. 4. (European Council on Computing in Construction, 2023).

[44] Bertin, I., Mesnil, R., Jaeger, J.-M., Feraille, A. & Le Roy, R. A BIM-based framework and databank for reusing load-bearing structural elements. *Sustainability* **12**, 3147 (2020).

[45] BAMB. Enabling a circular building industry. https://www.bamb2020.eu (2019).

[46] Bello, S. A., Yu, S., Wang, C., Adam, J. M. & Li, J. Review: deep learning on 3D point clouds. *Remote Sens.* **12**, 1729 (2020).

[47] López Iglesias, J. et al. Revision of automation methods for scan to BIM. In *Advances in Design Engineering* (Springer International Publishing, 2020).

[48] Maalek, R., Lichti, D. & Ruwanpura, J. Robust segmentation of planar and linear features of terrestrial laser scanner point clouds acquired from construction sites. *Sensors* **18**, 819 (2018). 29518062 10.3390/s18030819 PMC5876591

[49] Yuan, L., Guo, J. & Wang, Q. Automatic classification of common building materials from 3D terrestrial laser scan data. *Autom. Constr.* **110**, 103017 (2020).

[50] Turkan, Y., Bosché, F., T. Haas, C. & Haas, R. Tracking of secondary and temporary objects in structural concrete work. *Constr. Innov.* **14**, 145–167 (2014).

[51] Cha, Y.-J., Choi, W., Suh, G., Mahmoudkhani, S. & Büyüköztürk, O. Autonomous structural visual inspection using region-based deep learning for detecting multiple damage types: autonomous SHM using deep faster R-CNN. *Comput.-Aided Civ. Infrastruct. Eng.* **33**, 731–747 (2018).

[52] Clifford, B. & McGee, W.Cyclopean cannibalism: A method for recycling rubble. In ACADIA 2018: Recalibration. On imprecision and infidelity. Proceedings of the 38th Annual Conference of the Association for Computer Aided Design in Architecture (ACADIA) (pp. 404-413). Mexico City, Mexico. 10.52842/conf.acadia.2018.404

[53] Dimitrov, A. & Golparvar-Fard, M. Vision-based material recognition for automated monitoring of construction progress and generating building information modeling from unordered site image collections. *Adv. Eng. Inform.* **28**, 37–49 (2014).

[54] Xiao, K., Engstrom, L., Ilyas, A. & Ma, A. Noise or signal: the role of image backgrounds in object recognition. arXiv. https://arxiv.org/abs/2006.09994 (2021).

[55] Byers, B. S. & De Wolf, C. QR code-based material passports for component reuse across life cycle stages in small-scale construction. *J. Circ. Econ.* **1**, 1 (2023).

[56] Münsing, E., Mather, J. & Moura, S. Blockchains for decentralized optimization of energy resources in microgrid networks. In *2017 IEEE Conference on Control Technology and Applications (CCTA)*, 2017, 2164–2171 (IEEE, 2017).

[57] Hunhevicz, J. J. et al. Web3-based role and token data access: the case of building material passports. In *Presented at the EC3 Conference 2023, in Computing in Construction*, Vol. 4. (European Council on Computing in Construction, 2023).

[58] Hunhevicz, J. & De Wolf, C. Blockchain for a circular digital built environment. *Construction Blockchain Consortium, 2023 (CBC2023): IOT, Blockchain, and Smart Environments*, Hong Kong, China. 10.3929/ethz-b-000671734

[59] Excess Materials Exchange. (2020, January 21). Waste isn’t waste until we waste it. Medium. https://medium.com/@excessmaterialsexchange/waste-isnt-waste-until-we-waste-it-1bf128cf20d1

[60] Cao, J., Bucher, D. F., Hall, D. M. & Lessing, J. Cross-phase product configurator for modular buildings using kit-of-parts. *Autom. Constr.* **123**, 103437 (2021).

[61] Reflow. Co-creating circular and regenerative resource flow in cities. Project EU, 2022. https://reflowproject.eu (2022).

[62] Zhang, D. & Wang, X. C. Understanding many-to-many matching relationship and its correlation with joint response. *Transp. Res. Part B Methodol.* **108**, 249–260 10.1016/j.trb.2017.12.011 (2018).

[63] Durmisevic, E., Guerriero, A., Boje, C., Domange, B. & Bosch, G. Development of a conceptual digital deconstruction platform with integrated Reversible BIM to aid decision making and facilitate a circular economy. In *Proc. of the Joint Conference CIB W78 - LDAC 2021, 11-15 October 2021, Luxembourg*, 10 (2021).

[64] Rafner, J., Beaty, R. E., Kaufman, J. C., Lubart, T. & Sherson, J. Creativity in the age of generative AI. *Nat. Hum. Behav.* **7**, 1836–1838 (2023). 37985916 10.1038/s41562-023-01751-1

[65] Audry, S. *Art in the Age of Machine Learning* (MIT Press, 2021).

[66] Immanuel, K. *Artificial & Architectural Intelligence in Design—artificial-architecture* (Architecture and Sustainable Design, Singapore University of Technology and Design, 2020).

[67] Huang, J., Johanes, M., Kim, F. C., Doumpioti, C. & Holz, G.-C. On GANs, NLP and architecture: combining human and machine intelligences for the generation and evaluation of meaningful designs. *Technol. Des* **5**, 207–224 (2021).

[68] Schwarzkopf, V., Nolte, T. & De Wolf, C. Fostering creativity using AI towards a circular economy in architectural engineering design. In *International Association of Structures and Architecture (ICSA),**Special Session: Architectural Engineering Design and the Circular Economy* (Antwerp, 2025).

[69] Kaseb, Z. & Rahbar, M. Towards CFD-based optimization of urban wind conditions: comparison of Genetic algorithm, Particle Swarm Optimization, and a hybrid algorithm. *Sustain. Cities Soc.* **77**, 103565 (2022).

[70] Georgioudakis. M. & Plevris, V. A comparative study of differential evolution variants in constrained structural optimization. *Front. Built Environ*. 10.3389/fbuil.2020.00102 (2020).

[71] Tomczak, A., Haakonsen, S. M. & Łuczkowski, M. Matching algorithms to assist in designing with reclaimed building elements. *Environ. Res. Infrastruct. Sustain.* **3**, 035005 (2023).

[72] Brütting, J., De Wolf, C. & Fivet, C. The reuse of load-bearing components. *IOP Conf. Ser. Earth Environ. Sci.* **225**, 012025 (2019).

[73] Rossi, A. & Tessmann, O. From voxels to parts: hierarchical discrete modeling for design and assembly. In *ICGG 2018—Proceedings of the 18th International Conference on Geometry and Graphics* (eds Cocchiarella, L.) 1001–1012 (Springer International Publishing, 2019).

[74] Allner, L., Kroehnert, D. & Rossi, A. Mediating irregularity: towards a design method for spatial structures utilizing naturally grown forked branches. In *Impact: Design With All Senses* (eds Gengnagel, C. et al.) 433–445 (Springer International Publishing, 2020).

[75] Leder, S., Weber, R., Wood, D., Bucklin, O. & Menges, A. Distributed robotic timber construction: designing of in-situ timber construction system with robot-material collaboration. 10.52842/conf.acadia.2019.510 (2019).

[76] Brütting, J., Senatore, G. & Fivet, C. Optimization formulations for the design of low embodied energy structures made from reused elements. In *Advanced Computing Strategies for Engineering*, Vol. 10863, (eds Smith, I. F. C. & Domer, B.) in Lecture Notes in Computer Science, Vol. 10863, 139–163 (Springer International Publishing, 2018).

[77] Afshari, H., Hare, W. & Tesfamariam, S. Constrained multi-objective optimization algorithms: Review and comparison with application in reinforced concrete structures. *Applied Soft Computing.* **83**, 10563110.1016/j.asoc.2019.105631 (2019).

[78] Ng, M. S., Chen, Q., Hall, D. M., Hackl, J. & Adey, B. T. Designing for digital fabrication: an empirical study of industry needs, perceived benefits, and strategies for adoption. *J. Manag. Eng.* **38**, 04022052 (2022).

[79] Brandão, F., Paio, A. & Antunes, N. Towards a digitally fabricated disassemble-able building system: a CNC fabricated T-Slot joint. Computing for a better tomorrow - Proceedings of the 36th eCAADe Conference - Vol. 2 (eds Kepczynska-Walczak, A. & Bialkowski, S.) pp. 11-20 (Poland, 2018)

[80] Hua, H., Hovestadt, L. & Li, B. Reconfigurable modular system of prefabricated timber grids. *Comput.-Aided Des.* **146**, 103230 (2022).

[81] Kanters, J. Design for deconstruction in the design process: state of the art. *Buildings* **8**, 150 (2018).

[82] Sigrist, R., Rauter, G., Riener, R. & Wolf, P. Augmented visual, auditory, haptic, and multimodal feedback in motor learning: a review. *Psychon. Bull. Rev.* **20**, 21–53 (2013). 23132605 10.3758/s13423-012-0333-8

[83] Triantafyllidis, E., Mcgreavy, C., Gu, J. & Li, Z. Study of multimodal interfaces and the improvements on teleoperation. *IEEE Access* **8**, 78213–78227 (2020).

[84] Triantafyllidis, E. *Advancements in Sensory-Motor Perception and Biologically-Inspired Hierarchical Learning for Embodied Intelligence*. Doctoral Thesis, The University of Edinburgh, Edinburgh, UK (2024).

[85] Menny, T., Le Guirriec, S. & De Wolf, C. The butterfly matchmaking model for circular construction: Towards a digital matchmaking platform tailored to French policy. *Sustainable Production and Consumption* **49**, 130–143 10.1016/j.spc.2024.06.011 (2024).

[86] Circular Engineering for Architecture (CEA). SWIRCULAR: a Swiss Digital Circular Construction Ecosystem. *InnoSuisse Flagship* https://cea.ibi.ethz.ch/swircular.html (2024).

[87] Raghu, D. et al. Enabling component reuse from existing buildings. using google street view and machine learning to enhance building databases. In *Proc. of the 27th International Conference of the Association for ComputerAided Architectural Design Research in Asia (CAADRIA) 2022*, CAADRIA (2022).

[88] Raghu, D., Bucher, M. J. J. & De Wolf, C. Towards a ‘resource cadastre’ for a circular economy—urban-scale building material detection using street view imagery and computer vision. *Resour. Conserv. Recycl.* **198**, 107140 (2023).

[89] Çetin, S., Raghu, D., Honic, M., Straub, A. & Gruis, V. Data requirements and availabilities for material passports: a digitally enabled framework for improving the circularity of existing buildings. *Sustain. Prod. Consum.* **40**, 422–437 (2023).

[90] Byers, B., Cheriyamulla, S., Ewason, J., Hall, D. & De Wolf, C. Using engraved QR codes to connect building components to material passports for circular construction. In *Presented at the 2022 European Conference on Computing in Construction Ixia*. (Rhodes, Greece, 2022).

[91] RunwayML. Advancing creativity with artificial intelligence. Runway. https://runwayml.com/ (2024).

[92] Midjourney. https://www.midjourney.com/home?callbackUrl=%2Fexplore (2024).

[93] Huang, Y., Alkhayat, L., De Wolf, C. & Mueller, C. Algorithmic circular design with reused structural elements: method and tool. In *International fib Symposium—Conceptual Design of Structures 2021*, Sep. 457–468 (2021).

[94] Kantorovich, L. V. Mathematical methods of organizing and planning production. *Manag. Sci.* **6**, 366–422 (1960).

[95] Brütting, J., Senatore, G. & Fivet, C. Form follows availability—designing structures through reuse. *J. Int. Assoc. Shell Spat. Struct.* **60**, 257–265 (2019).

[96] Gordon, M. & De Wolf, C. Optimisation goals for efficient construction from reused materials towards a circular built environment. *Dev. Built Environ.* **19**, 100509 (2024).

[97] Ariza, I. et al. Lost and bound: adaptive detailing with robotic additive joining for reclaimed steel. In *Robarch, Beyond Optimization: Robotic Fabrication in Architecture, Art and Design* (Springer International Publishing, 2024).

[98] Wijewickrama, M. K. C. S., Rameezdeen, R. & Chileshe, N. Information brokerage for circular economy in the construction industry: a systematic literature review. *J. Clean. Prod.* **313**, 127938 (2021).

[99] Tseng, M.-L., Tan, R. R., Chiu, A. S. F., Chien, C.-F. & Kuo, T. C. Circular economy meets industry 4.0: can big data drive industrial symbiosis? *Resour. Conserv. Recycl.* **131**, 146–147 (2018).

[100] Esnaashary Esfahani, M., Rausch, C., Haas, C. & Adey, B. T. Prioritizing preproject planning activities using value of information analysis. *J. Manag. Eng.* **36**, 04020064 (2020).

[101] Bellini, A., Tadayon, A., Andersen, B. & Klungseth, N. J. The role of data when implementing circular strategies in the built environment: a literature review. *Clean. Environ. Syst.* **13**, 100183 (2024).

[102] Lausselet, C., Dahlstrøm, O. A., Thyholt, M., Eghbali, A. & Schneider-Marin, P. Methods to account for design for disassembly: status of the building sector. *Buildings* **13**, 1012 (2023).

[103] Gordon, M. et al. Automating building element detection for deconstruction planning and material reuse: a case study. *Autom. Constr.* **146**, 104697 (2023).

[104] Triantafyllidis, E., Acero, F., Liu, Z. & Li, Z. Hybrid hierarchical learning for solving complex sequential tasks using the robotic manipulation network ROMAN. *Nat. Mach. Intell.* **5**, 991–1005 (2023).

[105] Mildenhall, B. et al. NeRF: representing scenes as neural radiance fields for view synthesis. arXiv. 10.48550/arXiv.2003.08934 (2020).

[106] Wolpert, D. M., Diedrichsen, J. & Flanagan, J. R. Principles of sensorimotor learning. *Nat. Rev. Neurosci.* **12**, 739–751 (2011). 22033537 10.1038/nrn3112

[107] Billard, A. & Kragic, D. Trends and challenges in robot manipulation. *Science* **364**, eaat8414 (2019). 31221831 10.1126/science.aat8414

[108] Azhar, S., Ahmad, I & Sein, M. K. Action research as a proactive research method for construction engineering and management. *J. Constr. Eng. Manag.* **136**, 87–98 (2010).

[109] Riveiro, B., González-Jorge, H., Varela, M. & Jauregui, D. V. Validation of terrestrial laser scanning and photogrammetry techniques for the measurement of vertical underclearance and beam geometry in structural inspection of bridges. *Measurement* **46**, 784–794 (2013).

[110] Xiong, Z., Gordon, M., Byers, B. & De Wolf, C. Reality capture and site-scanning techniques for material reuse planning. In P*roceedings of the IASS/APCS 2022 Beijing Symposium: Sustainable Heritage Challenges and Strategies in the Preservation and Conservation of 20th Century Historic Concrete Shells* (Vol. 2022). International Association for Shell and Spatial Structures. 10.3929/ethz-b-000580345 (2022).

[111] Gordon, M. et al. Digitizing building materials for reuse with reality capture and scan-to-BIM technologies. In C. De Wolf, S. Çetin, & N. M. P. Bocken (Eds.), *A Circular Built Environment in the Digital Age* (pp. 41–55). 10.1007/978-3-031-39675-5 (Springer Nature, 2023).

[112] Gordon, M. *Mattersite*. Thesis Master Degree. Institute for Advanced Architecture of Catalonia, Vargas Calvo, R. (2021).

[113] Byers, B. S., Gordon, M., Iuorio, O. & De Wolf, C. Calculating embodied carbon for reused structural components with laser scanning. In Biondini, F. & Frangopol, D. M. (Eds.) *Life-Cycle of Structures and Infrastructure Systems*. (pp. 149–156) (CRC Press, 2023).

